# Tumor Necrosis Factor Disrupts Claudin-5 Endothelial Tight Junction Barriers in Two Distinct NF-κB-Dependent Phases

**DOI:** 10.1371/journal.pone.0120075

**Published:** 2015-03-27

**Authors:** Paul R. Clark, Richard K. Kim, Jordan S. Pober, Martin S. Kluger

**Affiliations:** Department of Immunobiology and Program in Vascular Biology and Therapeutics, Yale University School of Medicine, New Haven, Connecticut, United States of America; Emory University School of Medicine, UNITED STATES

## Abstract

Capillary leak in severe sepsis involves disruption of endothelial cell tight junctions. We modeled this process by TNF treatment of cultured human dermal microvascular endothelial cell (HDMEC) monolayers, which unlike human umbilical vein endothelial cells form claudin-5-dependent tight junctions and a high-resistance permeability barrier. Continuous monitoring with electrical cell-substrate impedance sensing revealed that TNF disrupts tight junction-dependent HDMEC barriers in discrete steps: an ~5% increase in transendothelial electrical resistance over 40 minutes; a decrease to ~10% below basal levels over 2 hours (phase 1 leak); an interphase plateau of 1 hour; and a major fall in transendothelial electrical resistance to < 70% of basal levels by 8–10 hours (phase 2 leak), with EC_50_ values of TNF for phase 1 and 2 leak of ~30 and ~150 pg/ml, respectively. TNF leak is reversible and independent of cell death. Leak correlates with disruption of continuous claudin-5 immunofluorescence staining, myosin light chain phosphorylation and loss of claudin-5 co-localization with cortical actin. All these responses require NF-κB signaling, shown by inhibition with Bay 11 or overexpression of IκB super-repressor, and are blocked by H-1152 or Y-27632, selective inhibitors of Rho-associated kinase that do not block other NF-κB-dependent responses. siRNA combined knockdown of Rho-associated kinase-1 and -2 also prevents myosin light chain phosphorylation, loss of claudin-5/actin co-localization, claudin-5 reorganization and reduces phase 1 leak. However, unlike H-1152 and Y-27632, combined Rho-associated kinase-1/2 siRNA knockdown does not reduce the magnitude of phase 2 leak, suggesting that H-1152 and Y-27632 have targets beyond Rho-associated kinases that regulate endothelial barrier function. We conclude that TNF disrupts TJs in HDMECs in two distinct NF-κB-dependent steps, the first involving Rho-associated kinase and the second likely to involve an as yet unidentified but structurally related protein kinase(s).

## Introduction

During acute inflammation, an increase in endothelial permeability (leak) above basal levels permits an exudate of large plasma proteins (e.g., fibrinogen and fibronectin) to form a provisional matrix in tissues upon which extravasating inflammatory leukocytes can migrate. This inducible (hyper)permeability is normally confined to post-capillary venule segments of the microcirculation [1,2] but in severe sepsis or in systemic inflammatory response syndrome (SIRS) may spread to the capillaries, resulting in widespread edema and organ failure [2–4]. Continuous capillaries are less prone than venules to leak because capillary endothelial cells (ECs) interconnect via tight junctions (TJs) organized around claudin-5 (CL5), whereas venular ECs primarily form adherens junctions (AJs) organized around VE-cadherin [5,6]. Capillary leak thus differs from venular leak by requiring disruption of TJs, a process poorly understood in ECs. This process could be an EC-intrinsic response to inflammatory mediators and/or arise from EC injury [7].

Individual cytokine-directed clinical trials have not led to effective therapies against sepsis probably because there are redundant mediators responsible for capillary leak in SIRS or severe sepsis. Despite such redundancy, analysis of the effects of a single mediator may reveal mechanisms that can be targeted to more broadly antagonize pathological processes. Two well recognized agents found elevated in SIRS and sepsis patients that have been extensively studied by many investigators are tumor necrosis factor (TNF, also called TNF-α) and IL-β [8,9] The injurious effects of TNF on ECs are mediated through TNF receptor (TNFR)-1, one of two different TNF receptors that may be expressed on microvascular ECs *in situ* [10], and TNFR1 occupancy by ligand results in *de novo* expression of various pro-inflammatory proteins, such as leukocyte adhesion molecules and chemokines, principally through NF-κB-dependent transcription [11]. Many of the same pro-inflammatory proteins are induced by IL-β binding to its receptor, also through NF-κB-dependent transcription [12]. The requirement for gene transcription and new protein synthesis in these responses imposes a delay of several hours before inflammation develops. TNF may also induce injury, i.e., EC death due to apoptosis or necroptosis, also after a delay of several hours [13], although TNF-mediated cell death is normally prevented in ECs by NF-κB-mediated synthesis of protective proteins [14]. EC overexpression of a mutated form of IκB that cannot be phosphorylated and thus not subject to polyubiquitinylation and degradation in response to TNF or IL-β, called “super repressor (SR)-IκB”, blocks TNF and IL-β induction of pro-inflammatory proteins. EC-specific expression of SR-IκB also reduces capillary leak *in vivo* in mouse models of sepsis [15]. However, the reduced leak caused by SR-IκB expression in mice could result either from inhibition of the intrinsic EC signaling responses that disrupt TJs, from the reduced leukocyte adhesion molecule expression that reduces interactions with neutrophils and monocytes that may cause EC injury, or from both processes combined.

Intrinsic responses of ECs have historically been analyzed *in vitro*, thereby eliminating the contribution of leukocytes. However, the choice and conditions of the culture system are critical. Most cultured ECs, including widely used human umbilical vein (HUV)ECs, form AJs but lack TJs despite expression of TJ proteins such as CL5. In contrast, human dermal microvascular (HDM)ECs, which form AJs upon reaching confluence, will, over a period of several days, develop frequent TJs that elevate transendothelial electrical resistance (TEER) to approximately two-fold greater levels than do HUVEC [16]. The elevated TEER and formation of TJs in this system requires expression of CL5. Furthermore, HDMEC monolayers that form TJs become resistant to calcium chelation [16], indicating that AJs are no longer required to maintain junctional integrity. HDMECs thus provide a model in which TJ disruption can be studied, but only in post-confluent cultures after TJs are allowed to mature. Here we report that in this model both TNF and IL-β can disrupt CL5-dependent TJs over a time course of several hours and that this response occurs in two distinct phases of leak, both of which require NF-κB activation. Furthermore, TJ disruption occurs without causing degradation of CL5 but is associated with both decreased co-localization of cortical actin filaments with CL5 and increased phosphorylation of myosin light chain. The later processes and phase 1 leak are mediated through the NF-κB-dependent activation of Rho-associated, coiled-coil containing protein kinases (commonly called ROCKs); phase 2 leak appears independent of ROCK 1 and 2 but is still blocked by small molecule inhibitors of ROCK, suggesting a role for an as yet unidentified but possibly related protein kinase(s). These observations using individual cytokines TNF and IL-β provide novel insights into the mechanism of leak in a TJ-dependent human endothelial barrier model system necessary for future studies with relevance to capillary leak using blood and bronchiolar fluids derived from SIRS and severe sepsis patients.

## Materials and Methods

### Antibodies and reagents

Immunocytochemistry was performed with polyclonal rabbit anti-CL5 (Invitrogen catalog #34-1600), goat anti-ICAM1 (R and D Systems, Minneapolis, MN; #BBA17), phalloidin-647 (Invitrogen) and DAPI (Invitrogen). Immunoblotting was performed with mouse mAbs anti-CL5 clone 4C3C2 (Invitrogen catalog #35-2500), anti-β-actin clone AC-74 (Sigma #A2228), anti-HSP90 clone 68 (BD Transduction Labs #610418); anti-myosin light chain 2 clone 19D3.1 (Millipore #MABT180) and anti-TNFR1 (Santa Cruz Biotechnology,#sc-8436); with purified rabbit polyclonal anti-phospho-(Thr18/Ser19) myosin light chain (Cell Signaling #3674); rabbit anti-ROCK1 clone C8F7 (Cell Signaling #4035), rabbit anti-ROCK2 clone D1B1 (Cell Signaling #9029), and rabbit mAb anti-MYPT1 Clone D6C1 (Cell Signaling #8574) and with goat anti-ICAM (R&D #BBA17). Cytokines used were recombinant human tumor necrosis factor-alpha (TNF; Invitrogen catalog #PHC3015) and bovine thrombin (GE Healthcare #27-0846-01). Inhibitors used were Bay11 (Tocris Bioscience #1743) for IκK-β, and Y-27632 (#688000) and H-1152 (#555550; dimethylfasudil) both from EMD Millipore for ROCK.

### EC cultures

HDMEC cultures used in this study were derived, by methods previously described [16,17], from adult human skin purchased from the tissue collection service of the Yale Department of Pathology or from the National Disease Research Interchange. All identifiers had been removed prior to receipt by laboratory personnel and no member of the laboratory group has had contact with any individual from whom skin was obtained. The Yale University Institutional Review Board, which is the Yale University Human Investigations Committee, has declared that isolation of cells from this tissue for use in our experiments does not constitute human subjects research and thus does not require informed consent. HDMECs resident in the papillary dermis were isolated by dermatome slicing the superficial most 0.5 to 0.7 mm of skin, followed by fine mincing and enzymatic digestion (Dispase, 50 U/ml; BD Biosciences) at 37°C until the epidermis removed easily from the underlying dermis. Released dermal cells were cultured on 10 μg/ml human plasma fibronectin-coated (Millipore) tissue culture plastic (BD Biosciences) in EGM2-MV growth medium (Lonza) that was replaced at 48 hour intervals. After colonies formed in primary culture, the cell populations were washed, and the substrate-adherent cells were resuspended with trypsin and immunoselected using anti-CD-31-biotin antibody followed by streptavidin-magnetic beads (both from Miltenyi Biotec). After replating, HDMECs were immunoselected a second time (which effectively depleted cultures of all non-EC cell types), serially passaged by trypsin resuspension onto 0.1% gelatin-coated tissue culture plastic (Falcon/Corning) and used between passages 4–7. Two or more different HDMEC isolates between passages 4–7 were used to confirm results in all experiments reported in this study.

### TEER measurements

**Fig 1:**
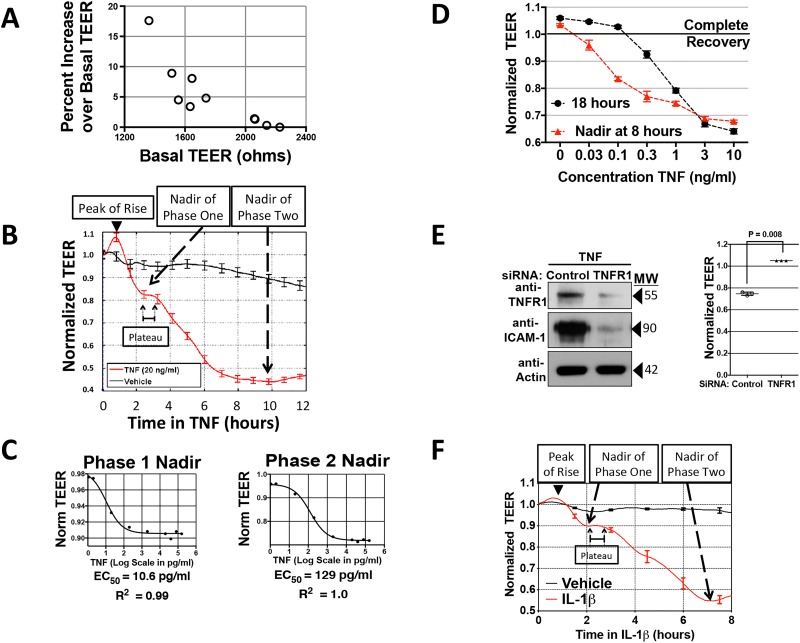
Kinetics and dose response of distinct changes to HDMEC barriers induced by TNF and IL-β. A: Relationship of the early TNF-induced TEER increase to basal TEER levels. A plot of the percent increase over basal TEER values (measured at the peak of the TNF-induced TEER increase, mean 0.7±0.01 hours; y-axis) vs. basal TEER (reported in ohms and read on a 96W20idf ECIS array; x-axis). TNF concentration was 20 ng/ml. The inverse correlation of basal TEER to the early TEER increase is statistically significant by a two-tailed Pearson analysis (p = 0.021 in 10 independent experiments). B: TNF induction of an early TEER rise and a bi-phasic TEER decrease. Labels indicate the peak of the early TEER increase and two distinct phases of TEER decrease (as nadirs to phases 1 and 2). X-axis, duration of incubation in 20 ng/ml TNF (units, hours); y-axis, in units of normalized TEER calculated as a ratio of TEER measurements taken post-TNF to the basal TEER level read before adding TNF, which is set at 1.0 (for a further explanation, please see Methods). The corrected basal TEER for this experiment was 63.7±1.2 Ω·cm^2^. n = 4,8 for vehicle, TNF. C: Relationship of TNF concentration to the decrease in TEER measured at the observed nadirs to phase 1 and phase 2. Example of data used to calculate EC_50_ values. Goodness of the non-linear regression curve fits are expressed as R-squared values. The corrected basal TEER for this experiment was 80.7± 0.8 Ω·cm^2^. D: Recovery of HDMEC barrier integrity relative to TNF concentration. TEER values show an inverse concentration-dependent recovery from phase 2 nadir levels (red trace) to pre-TNF basal levels in the continuous presence of TNF for 18 hours (black trace). The corrected basal TEER for this experiment was 57.5 ± 0.5 Ω·cm^2^. E: Effect of TNFR1 siRNA knockdown on TNF leak. Immunoblot analysis of siRNA silencing of TNFR1 expression confirmed by an inhibition of ICAM-1 expression (left) and ECIS analysis of the requirement for TNFR1 in TNF leak (right, y-axis TEER normalized to T_0_). The corrected basal TEER for this experiment was 75.6 ± 2.2 Ω·cm^2^ for TNFR1 siRNA-transfected HDMEC and 81.0 ± 1.2 Ω·cm^2^ for negative control siRNA-transfected HDMEC. Mean values are indicated by horizontal bars, n = 3,3) each at 10 hours of TNF at 0.8 ng/ml. MW, protein apparent molecular weight in kDa. F) Time course of discrete IL-β-induced changes in TEER (ECIS plot). Note that like TNF, IL-β (20 ng/ml) produced an initial small rise in TEER followed by two distinct phases of TEER decrease. The corrected basal TEER for this experiment was 72.0 ± 1.5 Ω·cm^2^. n = 3,3. Representative of 10 (A), 12 (B), 3 (C, D and F) or 2 (E) independent experiments with similar results.

Transendothelial electrical resistance (TEER) of HDMEC monolayers was assessed by electrical cell-substrate impedance (ECIS; Applied Biophysics) a technique for continuous measurement of barrier integrity [18]. Serially passaged HDMEC were plated at two-thirds confluence on fibronectin-coated 96-well gold electrode arrays (catalog # 96W20idf, or when high-throughput was not needed, the 8-well array catalog #8W10E+, both with polyethylene terephthalate; Applied BioPhysics). TEER measurements were obtained daily over 3 to 5 days to monitor increasing barrier integrity until HDMEC monolayers reached a plateau that coincided with maturation of tight junction morphology [16]. To initiate cytokine-induced changes in TEER, cytokine or vehicle control (EBM basal medium plus 0.1% endotoxin-free BSA) were introduced without replacing the growth medium at 48 hour post-feeding, a cell condition defined as quiescence. The TEER maximum obtained on quiesced HDMEC prior to adding cytokine is stated in the figure legend to each ECIS experiment in units of ohms per unit surface area (Ω·cm^2^) corrected by subtracting the reading from a cell-free well. This measurement is also referred to in the text as the basal TEER level. Figures illustrate normalized TEER values (where the value of 1.0 represents the basal TEER measurement immediately before adding cytokine). Normalization facilitates comparisons between experiments involving different HDMEC isolates because maximum obtainable basal barrier integrity varied among different HDMEC isolates used in this study and because HDMEC were plated on either 8W10E+ arrays or 96W20idf arrays, and the latter array type has double the electrode surface area per well and therefore halves the TEER level reported for an equivalent monolayer barrier. [The only exception to presentation of normalized TEER occurs in Fig. 1A in which we report the influence of different basal TEER levels on the early TNF-induced increase in barrier integrity. TEER values in Fig. 1A are reported in units of ohms corrected by subtracting the TEER value from a cell-free well and by subtracting TEER readings taken after addition of vehicle control (adding vehicle contributed to portion of the early TEER increase)]. The *n* values stated in the figure legends represent multiple replicate ECIS wells of individual experiments. HDMEC monolayer resistances were measured once every 60 seconds by application of a 1 μA constant AC current at 4000 Hz between a large and small electrode embedded in the chamber slide. Data was recorded by an ECIS Z-theta instrument controlled by a Dell personal computer ECIS equipped with ECIS software (Applied BioPhysics).

### Immunofluorescence microscopy and morphometric measurements

Treated HDMEC monolayers grown on fibronectin-coated glass were washed briefly and fixed in 95% ethanol for 30 minutes at 4°C. For two color immunofluorescence imaging, monolayers were incubated overnight in rabbit anti-CL5 antibody (Invitrogen, catalog #341600) and in some cases goat anti-ICAM-1 antibody (R and D Systems, #BBA17) diluted in TBS/0.2% Triton X-100/5% normal donkey serum. Alexa488 Donkey anti-Rabbit and Alexa594 Donkey anti-Goat secondary antibodies used to detect primary antibody and glass coverslips were mounted for immunofluorescence analysis in ProLong mounting media (Invitrogen). Randomly selected (5 images per experimental condition) fluorescence photomicrographs were collected using a Zeiss Axiovert fluorescence microscope and Plan-APOCHROMAT, 63x oil objective (Zeiss, Thornwood, NY) with a Hamamatsu ORCA-ER digital camera (Hamamatsu Photonics, Hamamatsu, Japan). The effect of TNF activation on CL5 junctional organization was quantified by an observer blinded to the treatment with ImageJ 1.48v software (http://imagej.nih.gov). Using the free-hand drawing and quantitation tools, we acquired measurements of the total paracellular junctional length and measurements of segments where CL5 immunostaining was “disorganized” (defined as conversion of condensed, contiguous and linear staining to a diffuse or segmented or sawtooth pattern). The percent of disorganized junctions was calculated as [length “disorganized” junctions ÷ total junctional length “total” junctions] × 100. Co-localization of anti-CL5 with actin staining was calculated using the Volocity 6.1.1 software co-localization tool with automatic thresholding based on Costes et al. [19] and expressed as a Pearson correlation co-efficient. For pooling of data from different siRNA experiments, the Pearson correlation co-efficients for each condition were normalized to the values for control siRNA without TNF treatment.

### Inhibition of cytokine-induced signaling

TNF-induced activation of NF-κB was inhibited pharmacologically with (2E)-3-[[4-(1,1-Dimethylethyl)phenyl]sulfonyl]-2-propenenitrile (Bay11; Tocris Bioscience) or by transduction with the murine S32/36A mutant SR-IκBα that had been inserted into the pLZRS retroviral vector downstream of an CMV promoter; the corresponding negative control was cells transduced by the same vector but without any insert [17]. Retroviral supernatants, produced by transfecting and selecting Phoenix packaging cells, were used for transducing HDMEC without selection. Transduction was highly efficient by inhibition of TNF-induced ICAM-1 expression assessed by immunoblot, IF microscopy and by flow cytometric analyses. ROCK-selective inhibitors Y-27632 or H-1152 resuspended in aqueous solution (water) were added to cell culture medium 30 minutes prior to addition of cytokines at final concentrations indicated in each figure legend (please see Discussion for a further explanation on concentration levels). HDMEC cultures were pre-treated with various pharmacologic inhibitors for 1 hour before adding TNF without changing growth medium. In all assays involving pharmacologic inhibitors, comparisons were made to replicate HDMEC cultures receiving an appropriate vehicle control (either DMSO dissolved in EBM basal medium for Bay11, or EBM for Y-27632 and H-1152) at volume and dilutions comparable to that of the drug.

### siRNA inhibition of gene expression

HDMECs plated on gelatin-coated (Sigma) 6-well plates at 50% confluence were spin-transfected with Oligofectamine (Invitrogen) complexed to siRNA sequences that specifically targeted TNFR1, ROCK-1, ROCK-2, both ROCK-1 and -2 or MYPT1. First, siRNA complexes of Oligofectamine (Invitrogen) at 50 μg/ml and siRNA at 100 nM were prepared in Opti-MEM I Reduced Serum Medium (Invitrogen) and then diluted five-fold in Opti-MEM to yield a final siRNA concentration of 20 nM. The siRNA-Oligofectamine were then added to HDMECs cultures and centrifuged at 1200 RCF for 5 minutes, followed by incubation for 2 hours at 37°C. Fresh medium (EGM-2MV, Lonza) was then added overnight and cells were re-transfected 24 hours later. After resting for 24 hours, HDMEC were then trypsinized and seeded into fibronectin-coated ECIS 8-chamber arrays (Applied BioPhysics, catalog #8W10E+) at 100,000 cells per well. Cell viability was assessed by re-plating and knockdown specificity assessed in comparison to a non-targeting siRNA control. siRNA sequences used were, as non-targeting siRNA control: 5'-UGGUUUACAUGUCGACUAA-3' (Dharmacon # D-001910-01-05); for targeting TNFR1: 5’-GUACAAGUAGGUUCCUUUGUU-3’ and 5'-UGGUUUACAUGUCGACUAA-3' (Dharmacon #D-005197-01 and #D-005197-02); for targeting MYPT: 5'-CAUCAGCUGGUGAUCGAUAtt-3’ and 5’-GCAGUACCUCAAAUCGUUUtt-3’ (Life Technologies # s9235 and # s9237); for targeting ROCK-1: 5’-CGGUUAGAACAAGAGGUAAtt-3’ and 5’-GGUUAGAACAAGAGGUAAAtt-3’ (Life Technologies # s12098 and s12097); and for ROCK-2: 5’-GGAGAUUACCUUACGGAAAtt-3’ and 5’-GAGAUUACCUUACGGAAAAtt-3’ (Life Technologies # s18161 and s18162).

### Immunoblot analysis of protein expression or of protein phosphorylation

For immunoblot analyses, HDMECs cultured in C12 plastic wells (BD Biosciences) were washed twice in ice cold PBS, scrape-harvested into Laemmli sample buffer [supplemented with 50 mM dithiothreitol (Sigma), 5 mM EDTA (Invitrogen), Phos-STOP (Roche), complete protease inhibitor and PefaBloc SC (both from Roche)] subjected to freeze-thaw and boiled for 5 minutes. Lysates were fractionated on SDS-PAGE gels, transferred at 4°C onto PVDF filters (Millipore), incubated in Blocking buffer (5% non-fat dry milk in 50 mM Tris-base, 150 mM NaCl, pH 7.4, 0.05% Tween-20), and bound with primary antibody overnight by rocking at 4°C. Blocking buffer containing 5% BSA in place of milk was used for blots involving anti-phospho-MLC. After incubation for 1 hour at room temperature with species-specific horse radish peroxidase-conjugated secondary antibody (Jackson ImmunoResearch Laboratories) detection of bound antibodies was performed with SuperSignal West Pico or Femto chemiluminescent substrates (Thermo Fisher Scientific). Immunoblot images display bands of interest and controls from the same contiguous immunoblots with background levels minimized by adjusting all pixels equally with ImageJ 1.48 software. Molecular weights in kDa calculated from protein size markers are included in each figure.

### Statistics

Data were analyzed with Prism 6.0e software. Significance of differences among groups was tested by one-way analysis of variance (ANOVA) followed by the Bonferroni post-test or by two-tailed t-tests that were paired or unpaired as fitting the experimental design. EC_50_ values for TNF were analyzed by non-linear regression (curve fit) using a least squares method. Data are expressed as a mean value ± SEM. P values of p <0.05 were considered significant.

## Results

### TNF-induced alterations in TEER occur in distinct phases

TNF, at a concentration of 20 ng/ml, caused an immediate but small rise in TEER that varied inversely relative to basal TEER levels as assessed by electrical cell-substrate impedance sensing (ECIS; Fig. 1A and B). The mechanism of this rise is unknown, but could relate to a TNF-mediated rise in the levels of cAMP [20] or of sphingosine-1-phosphate [21], second messengers that can increase TEER independently of NF-κB activation. This small TNF-induced increase over basal TEER levels (peak 4.5±1.6% at 0.7±0.01 h) was followed by a fall in TEER (designated as phase 1 leak) that stabilized at a nadir of 9.6±1.5% below basal TEER levels after 2.1±0.06 hours further incubation in TNF. This relatively stable plateau in TEER lasting 1.1± 0.09 hours was followed by a more significant decline in TEER, which reached a nadir at 67.6±2.8% of basal TEER at 8 to 10 hours of TNF incubation. These kinetics (Fig. 1B) were reproducible in 12 independent experiments with 8 separate HDMEC isolates, and the magnitude of each drop in TEER varied with TNF concentration. The EC_50_ TNF concentration for phase 2 leak (150±41 pg/ml) was five-fold higher than phase 1 leak (29.9±17 pg/ml; P<0.05 by paired two-tailed t-test in 3 independent experiments; Fig. 1C). TNF concentration affected the magnitude but not the kinetics of TEER change (Fig. 1C and data not shown). At concentrations near the phase 2 EC_50_ value, the decline in TEER recovered to pre-TNF levels by 18 hours of TNF, but recovery was incomplete at higher TNF concentrations (Fig. 1D). However, even these higher TNF concentrations did not induce cell detachment or produce nuclear alterations indicative of either apoptosis or necroptosis (data not shown). Silencing TNFR1 expression by siRNA abrogated both phases of TNF leak as well as induction of ICAM-1 protein expression (Fig. 1E), a target gene known to be TNFR1 dependent [22]. IL-β treatment also produced a biphasic fall in TEER of the same kinetics and magnitudes (Fig. 1F), and required greater EC_50_ concentrations of IL-1 β for phase 2 vs. phase 1 leak (5.5 vs. 0.24 ng/ml; 2.4 vs. 0.16 ng/ml) respectively and did not cause cell death in two different experiments (data not shown).

### Correlation of TNF-induced leak with disruption of TJs

**Fig 2:**
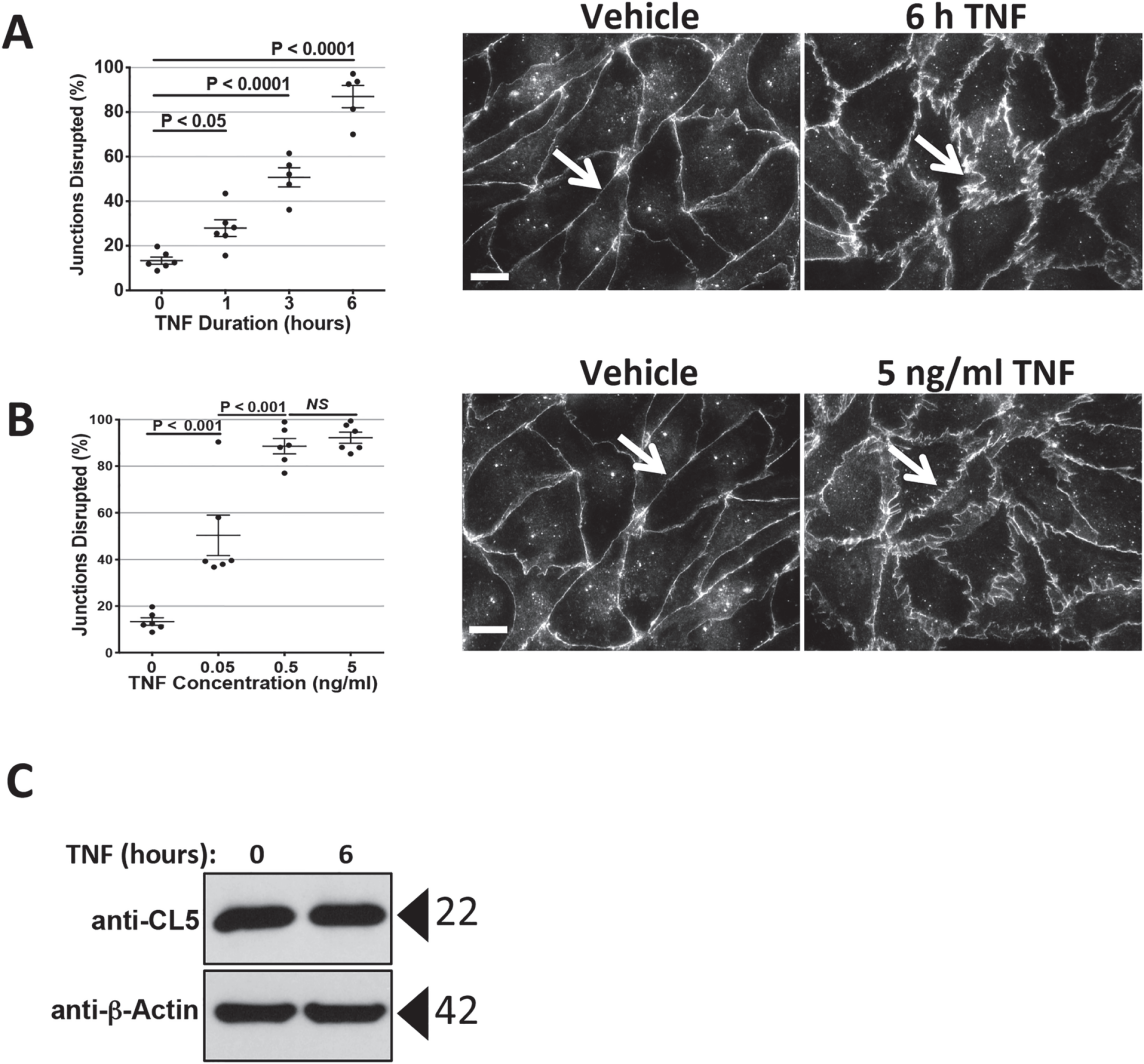
Morphometric analysis of TNF-induced disruption of CL5 junctions. Left: A) Time course of TNF (10 ng/ml)-induced CL5 disruption and B) dose response of TNF-induced CL5 disruption (measured at 6 hours of TNF in the same experiment) assessed as described in the Methods. Lines are mean ± SEM. p values computed by one-way ANOVA, Bonferroni post-tests are shown. Right: Examples of the immunofluorescence microscopy images assessed at a selected time point in (A) and TNF concentration in (B). Arrows compare CL5 junctional staining that changes from compact and contiguous to diffuse and disrupted after 6 hours in the presence of TNF. Scale bars, 15 μm. C) Effect of 6 hours TNF at 0.8 ng/ml on CL5 expression levels by immunoblot. Representative of 2 (A and B) or 3 (C) independent experiments with similar results.

We used immunofluorescence microscopy to correlate TNF effects on TEER with morphological changes in the pattern of CL5 at junctions. As previously shown, newly confluent HDMEC monolayer barriers are initially TJ-independent because they start synthesizing CL5 protein only after reaching confluence. The CL5 staining pattern observed by IF microscopy gradually coalesces by day 5 post-confluence to become a tight, continuous peripheral band as in parallel, TEER rises [16]. TNF treatment disrupts the condensed contiguous staining pattern, producing a diffuse segmented and “sawtooth” pattern discernible by 2–3 hours and more pronounced by 6 h, consistent with the kinetics of the fall in TEER. These changes are sensitive to TNF concentration and are maximally altered by 0.5 ng/ml TNF (at 6h), approximating the EC_50_ for phase 2 leak computed from ECIS measurements (Fig. 2A and B). No changes in whole cell HDMEC expression of CL5 was observed by Western blotting through the 6 hour time point (Fig. 2C) suggesting that the altered IF pattern that correlates with leak reflects a reorganization and not a degradation of CL5.

### **TNF leak requires NF-**κ**B activation**

**Fig 3:**
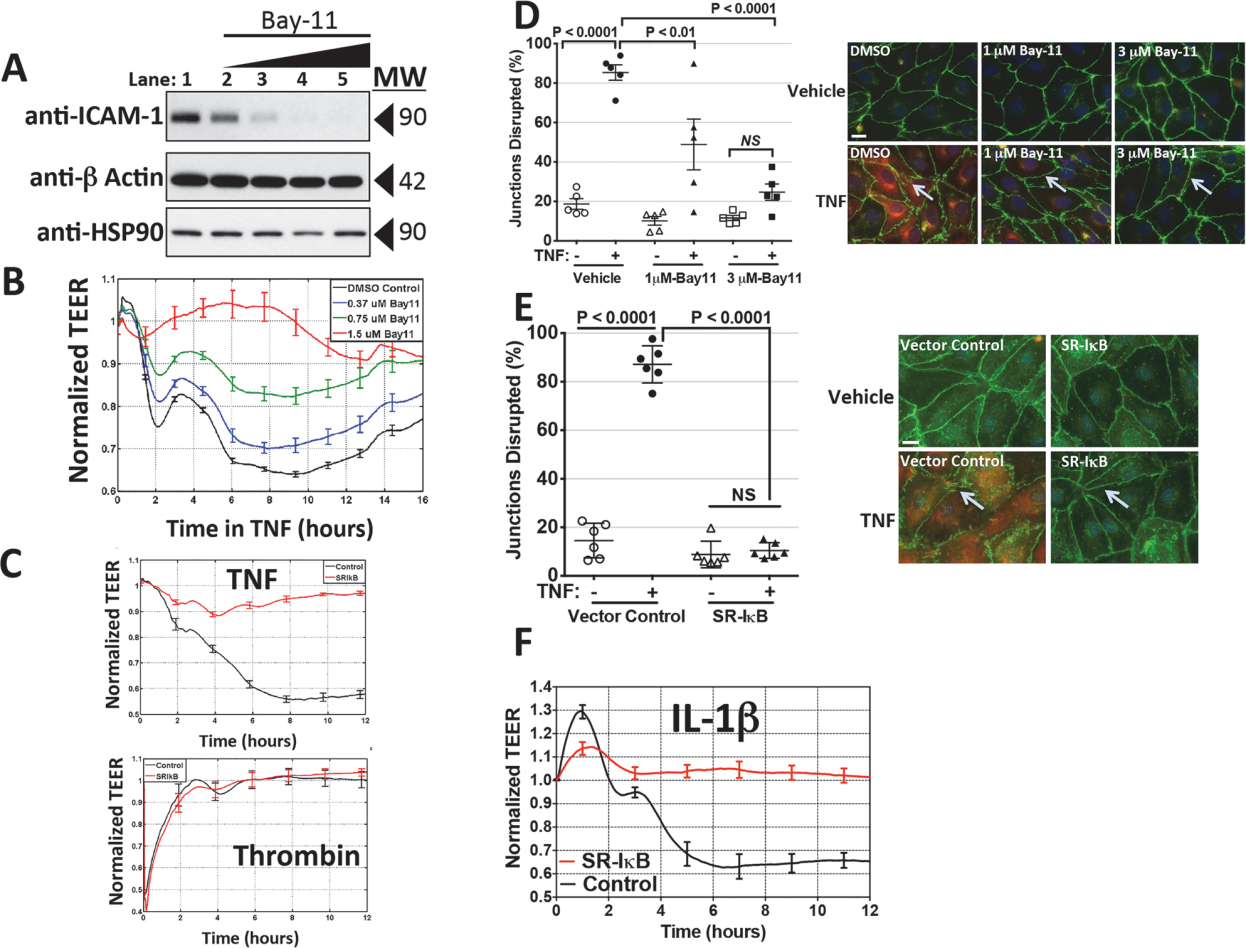
Requirement of NF-κB activation for cytokine-mediated reductions in TEER. A: Concentration-dependent inhibition of induction of ICAM-1 expression by IκK-β-inhibitor Bay11, confirming the effect of Bay11 on TNF induction of NF-κB-dependent genes. Conditions are lane 1, DMSO Control; lanes, 2, 3, 4 and 5, Bay11 at 0.375, 0.75, 1.5 and 3.0 μM, respectively all after 0.8 ng/ml TNF for 16 hours. B: Effect of Bay11 concentration on the TNF-induced TEER decrease. ECIS analysis. X-axis: Duration of 0.8 ng/ml TNF treatment. Y-axis: TEER (ohms) normalized to basal barrier level prior to addition of TNF. TEER levels were not affected by these concentrations of Bay11 in the absence of TNF (not shown). The corrected basal TEER for this experiment was 69.9 ± 1.3 Ω·cm^2^. n = 6,6,6,6. C: Effects of SR-IκB dominant negative overexpression on HDMEC barrier responses. Upper panel: A time course of TNF treatment (1 ng/ml for 12 h) in control-transduced (black trace) and SR-IκB-transduced (red trace) HDMEC. n = 4,4. Lower panel: A time course of thrombin (1 U/ml for 12 h) in control-transduced (black trace) and SR-IκB-transduced (red trace) HDMEC. The corrected basal TEER for this experiment was 72.4 ± 0.9 Ω·cm^2^ for SR-IκB-transduced HDMEC and 80.0 ± 0.7 Ω·cm^2^ for vector control-transduced HDMEC. n = 3,3. Note that the phase 1 and phase 2 decreases initiated by TNF are markedly inhibited in SR-IκB-relative to control-transduced HDMEC but that thrombin-induced TEER decreases are similar in the same control- and SR-IκB-transduced HDMEC lines. D: Effects of Bay11 (used at 1 or 3 μM as labeled) on disruption of CL5 staining by 6 hours of TNF at 10 ng/ml. Morphometric measurements of TNF-induced disruption of CL5 junctional staining as described in the Methods (left). Immunofluorescence images representative of those used to assess the extent of disruption (right). Anti-CL5 (green), and anti-ICAM (red). Scale bar, 15 μm. E: Effects of SR-IκB transduction on disruption of CL5 staining by 6 hours of TNF at 10 ng/ml. Morphometric measurements (left) and representative immunofluorescence (right) images as in (D). Anti-CL5 (green), and anti-ICAM (red). Scale bar, 15 μm. Note that Bay 11 and SR-IκB each prevented the induction of ICAM-1 as well as the induction of a disrupted pattern of anti-CL5 immunofluorescence staining (arrows in D and E) by TNF. F: Effects of SR-IκB dominant negative overexpression on IL-1β-leak (ECIS). Note that IL-1β over a 12 hour time course was ineffective at decreasing TEER in SR-IκB-transduced HDMEC. The corrected basal TEER for this experiment was 64.5 ± 2.8 Ω·cm^2^ for SR-IκB-transduced HDMEC and 59.6 ± 1.1 Ω·cm^2^ for vector control-transduced HDMEC. n = 3,3. Representative of 3 (A, B and C) or 2 (D, E and F) independent experiments with similar results.

The kinetics of TNF- or IL-β-induced leak and changes in CL5 organization require several hours, suggestive of a need for new protein synthesis requiring NF-κB activation. Bay11 a pharmacological inhibitor of IκB kinase-β that prevents canonical NF-κB signaling in response to TNF or IL-β, inhibited TNF leak in a dose-dependent manner up concordant with inhibition of TNF-induced ICAM-1 expression and consistent with the observation that most TNF and IL-1β-induced proteins depend upon NF-κB activation (Fig. 3A and B). Higher concentrations of the drug proved toxic to HDMEC monolayers, leading to a collapse of barrier function in the absence of TNF. Therefore, to confirm these data acquired by use of an agent with a potential for toxicity, we also inhibited NF-κB-dependent gene induction in HDMEC by overexpressing SR-IκB. Whereas the barrier formed by control vector-transduced HDMEC remained responsive to TNF, SR-IκB-transduced HDMEC were unresponsive to TNF but remained fully sensitive to thrombin-induced loss of barrier function as measured by ECIS (Fig. 3C; Unlike TNF, thrombin induces a rapid, transient calcium-dependent fall in TEER [23,24]. Moreover, Bay11 addition and SR-IκB over-expression each prevented TNF-induced disruption of the junctional CL5 staining pattern readily observable in DMSO-treated or control-transduced HDMEC, respectively (Fig. 3D and E). Finally, SR-IκB-transduced HDMECs were also resistant to IL-1β-induced phase 1 and phase 2 leak (Fig. 3F).

### TNF leak correlates with actin cytoskeletal reorganization

**Fig 4:**
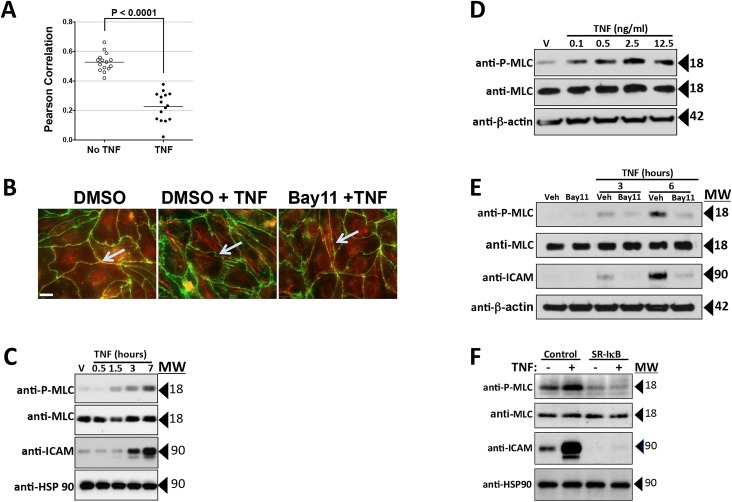
Effects of TNF on the actin cytoskeleton and MLC phosphorylation. A) Analysis of the effects of TNF on actin/CL5 co-localization. Post-confluent HDMEC monolayers immunostained with anti-CL5 and phalloidin-stained for actin were imaged by fluorescence microscopy and analyzed for co-localization as described. Actin/CL5 co-localization was lost after TNF treatment for 8 hours at 0.8 ng/ml, resulting in a statistically significant difference in the Pearson correlation co-efficient (y-axis) by two-tailed t-test. B) Immunofluorescence microscopy representative of the data in Fig. 4. Co-localization of the cortical actin cytoskeleton with junctional CL5 in DMSO control HDMEC (arrow in left panel; phalloidin staining, red; anti-CL5, green) is dissociated by TNF (center panel), a change prevented by Bay11 (right panel). Scale bar, 15 μm. C) Time course of TNF-induced changes in MLC (Thr18/Ser19) phosphorylation and ICAM-1 levels measured by immunoblotting with controls for total MLC and for β-actin. TNF treatment for the times indicated was at 10 ng/ml. D) Dose response of TNF-induced changes in phospho-MLC levels assessed by immunoblot analysis. HDMEC lysates were harvested at 6 hours of TNF. E) Effect of Bay11 on changes in P-MLC levels induced by TNF. Bay-11 was used at a 3 μM concentration. F) Effect of SR-IκB on changes in P-MLC levels induced by TNF. In E and F) TNF treatment was for 6 hours at 0.8 ng/ml. Note that Bay11 and SR-IκB each inhibit TNF-induced increases in ICAM-1 protein levels as well as MLC phosphorylation. Representative of 2 (B, E) or 3 (C, D, F) independent experiments with similar results.

**Fig 5:**
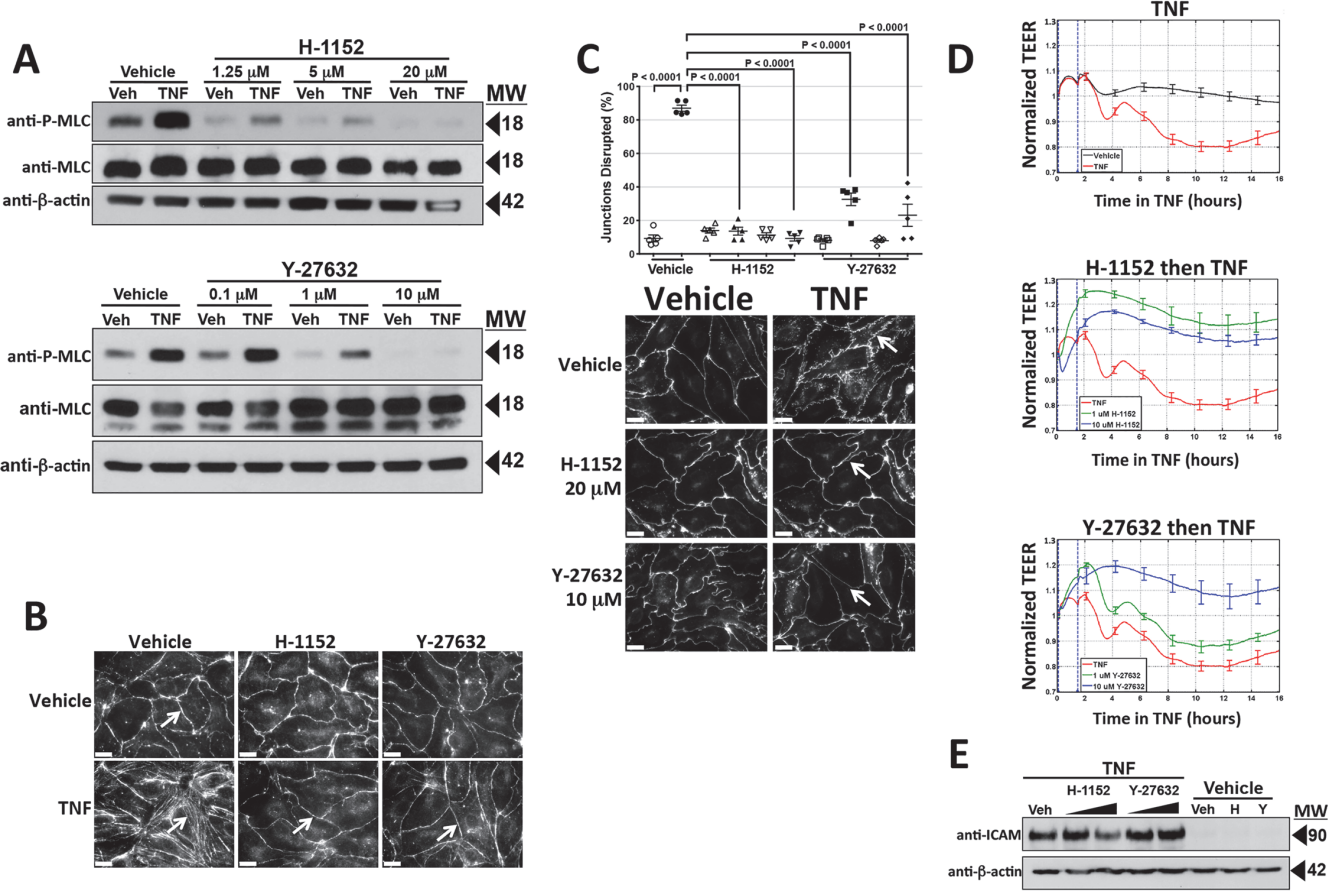
Effects of ROCK inhibitors H-1152 and Y-27632 on TNF-induced MLC phosphorylation, actin and CL5 reorganization and the fall in TEER. A) Immunoblot analysis of the effects of H-1152 (top) or Y-27632 (bottom) concentration on induction of MLC phosphorylation at 6 hours of 0.8 ng/ml TNF. B) Fluorescence microscopy analysis of the effects of H-1152 or Y-27632 (at 10 μM concentrations) on TNF-induced actin re-organization. Actin visualized by phalloidin staining. Note that changes to the peripheral pattern of cortical actin at 6 hours of 0.8 ng/ml TNF treatment are inhibited by H-1152 and by Y-27632 (arrows). Scale bar, 15 μm. C) Top: Morphometric analysis of the effects of H-1152 (at concentrations of 2 or 20 μM, triangles and inverted triangles, respectively), and Y-27632 (at concentrations of 1 or 10 μM, squares and diamonds) on disruption of CL5 junctional staining at 6 hours of 0.8 ng/ml TNF. Open symbols, no TNF, closed symbols, plus TNF. Below: Examples of the morphometrically assessed immunofluorescence microscopy images. Note that CL5 staining disorganized by TNF in vehicle control appears condensed and contiguous in the presence of both ROCK inhibitors (arrows). Scale bar, 15 μm. D) Effects of H-1152 and Y-27632 on the phase 1 and phase 2 TEER decreases induced by TNF. Starting at T_0_, HDMEC plated on ECIS 96-well arrays received a 1 hour pre-treatment with vehicle (top panel, n = 4, 4) or with a ROCK inhibitor, either H-1152 (at 1 or 10 μM, middle panel, n = 6, 6) or Y-27632 (at 1 or 10 μM, bottom panel, n = 6, 6). One hour later 0.8 ng/ml TNF or (top panel only) vehicle was added, indicated by the vertical dashed lines. The corrected basal TEER for this experiment was 67.8 ± 0.4 Ω·cm^2^. E) Immunoblot analysis of the effects of H-1152 and Y-27632 on TNF-induced ICAM expression. Protein lysates were from HDMEC pre-treated with H-1152 (1 or 10 μM) or Y-27632 (1 or 10 μM) then TNF, 0.8 ng/ml for 6 h. Representative of 4 (A), 3 (B, D, E) and 2 (C) independent experiments with similar results.

**Fig 6:**
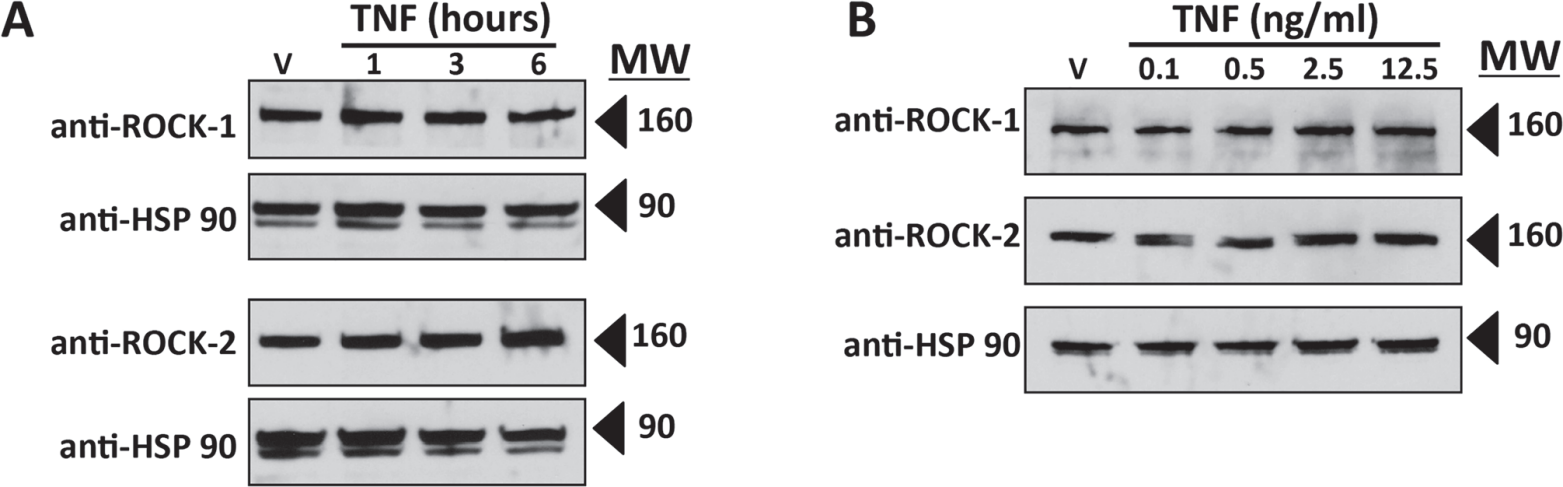
Effects of TNF on ROCK expression. A) Time course of TNF-induced effects on levels of ROCK-1 and ROCK-2 expression measured by immunoblotting. TNF concentration, 0.8 ng/ml. B) Dose response of TNF-induced effects on ROCK-1 and ROCK-2 expression levels assessed at 6 hours of TNF by immunoblot analysis. Note that over the time course and the range of TNF concentrations tested the ROCK-1 and ROCK-2 expression levels were essentially unchanged. Anti-HSP90, gel loading control.

Thrombin-mediated leak has been shown to involve actin cytoskeleton reorganization and isometric tension mediated through phosphorylation and activation of MLC [25]. The cortical actin cytoskeleton of HDMECs is also re-organized by TNF [17]. Cortical actin filaments, distinctly visible and co-localized with CL5 in post-confluent cells, lost CL5 co-localization upon TNF treatment (Fig. 4A) which was inhibited by Bay11 (Fig. 4B). TNF also increased phospho-MLC (but not total MLC) expression levels with kinetic and concentration responses consistent with the onset of TNF leak (Fig. 4C and D). Furthermore, TNF-induced phosphorylation of MLC was blocked by treatment with Bay11 or by transduction with SR-IκB (Fig. 4E and F). MLC phosphorylation may be mediated by several distinct pathways, but the two best understood involve calcium/calmodulin-activation of MLC kinase (MLCK), and RhoA/ROCK-inhibition of MLC phosphatase through phosphorylation of its MYPT1 subunit [25–27]. As we have not observed any changes in cytosolic calcium as a result of TNF treatment (unpublished work, JSP), and because TNF has previously been reported to influence ROCK [26], we focused on this second pathway. Two different selective inhibitors of ROCK, Y-27632 and H-1152, each blocked TNF-induced MLC phosphorylation, diminished cortical actin filament co-localization with CL5, CL5 redistribution and the fall in TEER (Fig. 5A-D) without evidence of toxicity. The concentrations of these drugs required to block these responses (see Discussion) did not inhibit induction of ICAM-1 by TNF, indicating that ROCK activation occurs downstream of NF-κB-mediated protein induction (Fig. 5E). TNF treatment did not affect the levels of ROCK 1 or ROCK 2, the isoforms of this enzyme expressed by HDMECs (Fig. 6).

**Fig 7:**
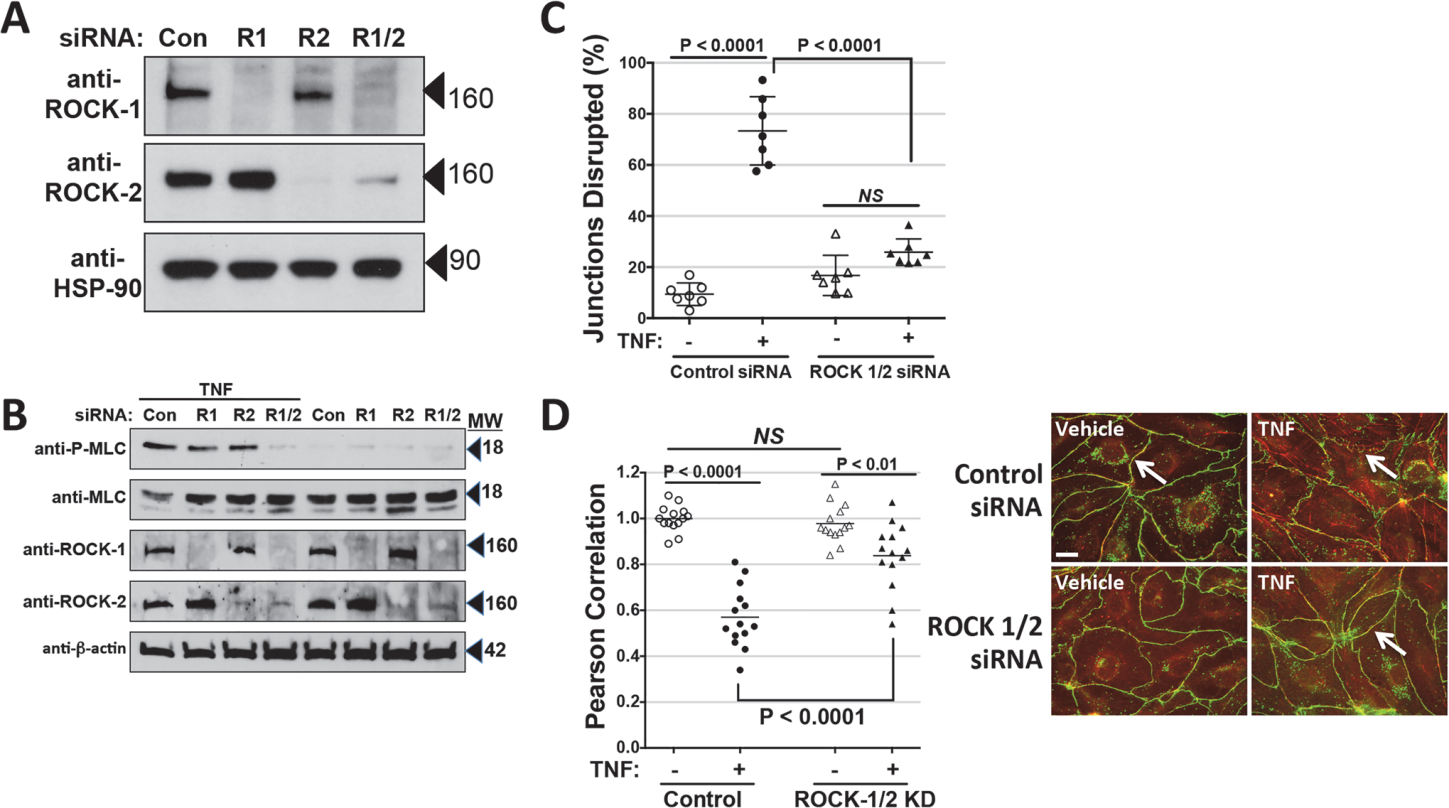
Effects of ROCK-1, -2 siRNA knockdown on MLC phosphorylation and the actin cytoskeleton. A) Immunoblot analysis of siRNA knockdown of ROCK-1 (R1), ROCK-2 (R2) or ROCK-1 and -2 (R1/2) expression. B) Immunoblot analysis of the effects of siRNA knockdown of ROCK expression on MLC phosphorylation at 6 hours of 0.8 ng/ml TNF. Note that MLC-phosphorylation was inhibited most effectively in lysates from TNF-treated ROCK-1/2 KD HDMEC. C) Morphometric analysis of the effects of ROCK-1/2 KD on disruption of CL5 junctional staining induced by 6 hours of 0.8 ng/ml TNF. D) Analysis of the effects of ROCK-1/2 KD on loss of actin/CL5 co-localization. Silencing of ROCK1/2 expression was effective at inhibiting the loss of actin/CL5 co-localization at 6 hours of 0. 8 ng/ml TNF treatment as measured by differences in Pearson correlation co-efficients (y-axis) analyzed by one-way ANOVA with a Bonferroni post-test. Data pooled from two independent experiments in which n = 7,7,7,7. Right: Examples of the immunofluorescence microscopy images assessed in C and D. Note that the TJ disruption and the loss of co-localization induced by TNF in control HDMEC was inhibited in ROCK-1/2 KD HDMEC (arrows). Scale Bar, 15 μm. Representative of 3 (A, B) and 2 (C) independent experiments with similar results.

**Fig 8:**
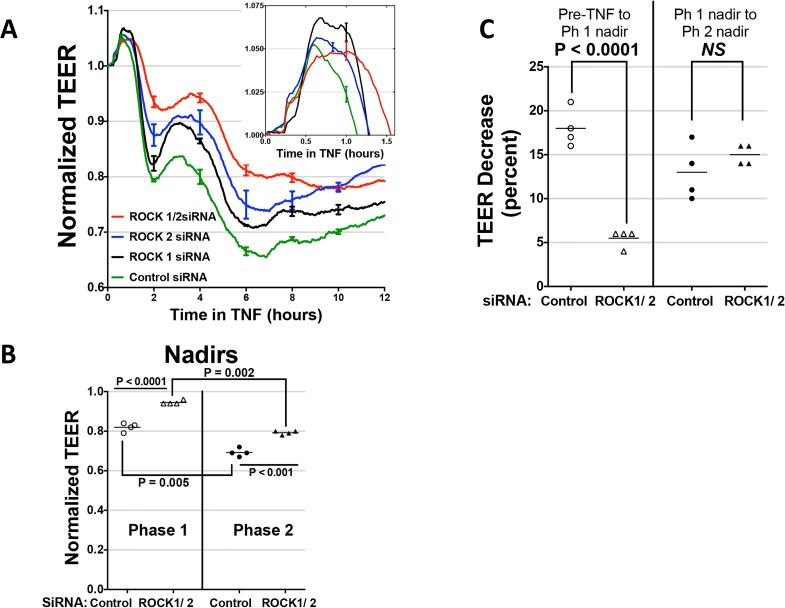
Distinct requirements for ROCK-1/2 expression in phase 1 and phase 2 leak. A) Effects of ROCK-1, ROCK-2 or ROCK1/2 siRNA knockdown on the time course of TEER decrease induced by 0.8 ng/ml TNF. Note that the combined ROCK-1/2 KD was more effective than either single knockdown at inhibiting TNF leak. Inset: The early rise in TEER, enlarged to highlight error bars indicating the statistical variation of this increase. The corrected basal TEER for this experiment was 63.8 ± 1.2 Ω·cm^2^ for ROCK-1 siRNA-transfected HDMEC, 67.2 ± 0.9 Ω·cm^2^ for ROCK-2 siRNA-transfected HDMEC, 68.9 ± 1.0 Ω·cm^2^ for combined ROCK-1, and -2 siRNA-transfected HDMEC, and 71.8 ± 0.8 Ω·cm^2^ for negative control siRNA-transfected HDMEC. ECIS analysis, n = 4,4,4,4. B) Effect of ROCK-1/2 KD on TEER level at the nadirs to phase 1 and phase 2 of TNF leak. Note that the TNF-induced decrease from T_0_ to the nadir of phase 1 was less in ROCK-1/2 KD than in siRNA control HDMEC (open triangles and circles, respectively). In addition, TEER levels decreased from the nadir of phase 1 to the nadir of phase 2 TNF leak in both ROCK-1/2 KD and siRNA control HDMEC (closed triangles and circles, respectively). The differences indicated were statistically significant by paired two-tailed t-tests. C) Comparison of the TEER decreases occurring in phase 1 vs. in phase 2 TNF leak by ROCK1/2 KD and by control HDMEC. Note that in comparison to control HDMEC, ROCK-1/2 KD inhibited the phase 1 TEER decrease (from T_0_ when TNF was added until the nadir of phase 1), but did not inhibit the TEER decrease from the nadir of phase 1 to the nadir for phase 2. Statistically significant where indicated by an unpaired two-way t-test. TNF concentration, 0.8 ng/ml in B) and C). Data in (A) is representative of 4 independent experiments with similar results and data in (B) and (C) show pooled data from the same 4 independent experiments.

**Fig 9:**
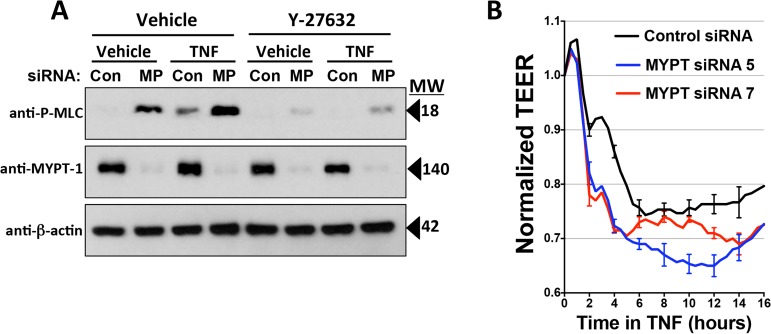
Analysis of requirement for MYPT1 expression in TNF-induced MLC phosphorylation and leak. A) Immunoblot analysis of MLC phosphorylation in lysates of MYPT1 KD HDMEC. In the absence of TNF, phospho-MLC expression levels were higher in lysates from MYPT1 siRNA-transfected HDMEC than in lysates of siRNA control. TNF treatment for 6 hours at 0.8 ng/ml further increased levels of phospho-MLC expression in MYPT1 KD HDMEC, but MYPT1 knockdown did not limit inhibition of TNF-induced MLC phosphorylation by 10 μM Y-27632. Con, siRNA control lysate; MP, MYPT1 siRNA lysate. B) ECIS analysis of TNF leak in MYPT1 KD HDMEC. Compared to control-transfected HDMEC, MYPT1 KD did not inhibit leak induced by TNF at 0.8 ng/ml. Basal levels of TEER in MYPT1 KD HDMEC were decreased to 69 ± 0.3% that of control siRNA (data not shown). The corrected basal TEER for this experiment was 65.3 ± 1.9 Ω·cm^2^ for MYPT siRNA 5-transfected HDMEC, 41.6 ± 1.5 Ω·cm^2^ for MYPT siRNA 7-transfected HDMEC, and 42.1 ± 0.8 Ω·cm^2^ for negative control siRNA-transfected HDMEC. n = 3,3,3. Representative of 2 (A, B) independent experiments with similar results.

The effects of Y-27632 and H-1152 suggest that ROCK activation is required for TNF effects on MLC phosphorylation and leak. However, both of these drugs can inhibit several other structurally related protein kinases. To verify the role of ROCK, we turned to an siRNA approach. HDMEC express two different ROCK isoforms, so we performed several experiments in which ROCK-1, ROCK-2 or both ROCK-1 and -2 expression were silenced. TNF-induced MLC phosphorylation was partially inhibited by siRNA KD of ROCK-1 or of ROCK-2, and nearly completely inhibited in HDMEC silenced for expression of both ROCK-1 and -2 (Fig. 7A and B). ROCK-1/2 KD HDMEC were largely protected from TNF effects on TJ disruption and, to a statistically significant but lesser extent, on CL5 co-localization with cortical actin (Fig. 7C and D). Combined knockdown also inhibited TNF leak more effectively than did single knockdown of ROCK-1 or -2. However, combined ROCK-1/2 siRNA KD inhibited TNF-induced leak less effectively than H-1152 or for Y-27632 (at 10 μM but not at the lower 1 μM concentration). Moreover, ROCK-1/2 KD only reduced the magnitude of phase 1 but not phase 2 TNF leak (Fig. 8A-C). Finally, to test if the effects of ROCK activation in TNF-induced MLC phosphorylation occur through inhibition of myosin phosphatase activity, we silenced MYPT1 expression in HDMECs using siRNA. This treatment dramatically increased basal levels of phospho-MLC expression, but levels were increased substantially further by TNF treatment, suggesting that ROCK-dependent MLC phosphorylation is MYPT-independent. siRNA knockdown of MYPT1 expression in HDMEC did not limit inhibition of TNF-induced phospho-MLC expression by Y-27632, providing further evidence that the ROCK pathway for MLC phosphorylation activated by TNF was at least partly independent of MYPT1. Concordantly, MYPT-1 knockdown did not limit the TNF-induced TEER decrease in comparison to control transfectants (Fig. 9). Cumulatively, these experiments suggest that phase 1 leak by TJ-dependent HDMEC monolayers is mediated by NF-κB-dependent activation of both ROCK-1 and ROCK-2, and that activated ROCK-1 and -2 contribute to MLC phosphorylation, disassociation of CL5 from cortical actin filaments and disruption of CL5 TJs. However, phase 2 leak, while also dependent upon NF-κB activation and blocked by (what are widely believed to be) ROCK-kinase selective inhibitors, appears to occur independently of ROCK-1 and 2 expression, MLC phosphorylation or MLC-dependent changes in the actin cortical cytoskeleton.

## Discussion

Enhanced endothelial leak through post-capillary venules is a crucial component of inflammation that involves disruption of VE-cadherin organized AJs. There have been many studies analyzing this process as a response to inflammatory mediators, such as TNF or IL-β, *in vitro*. However, endothelial leak through capillaries, which is associated with SIRS or severe sepsis, involves disruption of CL5-organized TJs, structures not found in venules or in most EC culture systems. This process of capillary leak therefore differs and is much less well understood. We recently reported that cultured HDMECs can form TJs when kept at post-confluence and that TEER in post-confluent HDMECs is dependent on CL5 expression at TJs. Here we use this model system to analyze how inflammatory cytokines, especially TNF, affect these structures. Our study makes several novel and unexpected findings. First, the response of TNF (and IL-β)-induced leak in HDMECs is complex, involving two distinct phases with different EC_50_ values for these cytokines. Second, both phases are dependent upon activation of NF-κB. Third, the initial fall in barrier function (phase 1 leak) correlates temporally with disruption of CL5 junctional continuity and with onset of MLC phosphorylation, which also are NF-κB-dependent and as shown by siRNA knockdown, are dependent on activation of ROCK-1 and -2. Interestingly, the TNF-induced, ROCK-mediated phosphorylation of MLC appears largely independent of MYPT1. Fourth, the second phase of TNF-induced leak, while blocked by selective inhibitors of ROCK, are largely unaffected by siRNA combined knockdown of ROCK-1 and -2, implying the involvement of other structurally related but distinct kinase(s).

ECs are perhaps the major systemic target of TNF, which triggers *de novo* gene expression by activating the master transcriptional regulator NF-κB. These pathways have been studied intensively but less so with regard to the effect of TNF on EC barrier function. TNF invariably triggers two distinct phases of leak. Both phases of TNF-induced leak in HDMEC cannot be attributed to apoptosis or necroptosis based on the absence of nuclear condensation or fragmentation, respectively. Further evidence against irreversible injury is that there is a recovery to basal barrier levels at effective TNF doses within 18 hours. The NF-κB-dependent programs, which remain to be identified, must be highly regulated in order to produce the statistically uniform kinetics and leak nadirs described. The explanation we propose for how the same cytokine, acting through the same receptor (TNFR1), can initiate two processes with different kinetics and concentration dependency is that the more rapid process involves transcriptional events dependent solely upon preformed transcription factors (as seen in early induction of E-selectin) whereas the slower process requires de novo synthesis of additional transcription factors (as seen in the later induction of VCAM-1) [28,29]. Similarly, the differing EC_50_ values of TNF or IL-β required for phase 1 vs. phase 2 leak may be explained by different signal strengths being required for the activation of different transcription factors [30].

Our results differ from a previous report describing immediate rapid changes in the HDMEC barrier by TNF and IL-β involving the small G protein Arf6 that are independent of NF-κB-activation [31]. However, a second publication by the same group reveals two key differences in our models: a) the effects they observe were made using HDMEC monolayers at time points that likely precede TJ-dependent barrier formation and at which AJs provide barrier integrity; and b) VE-cadherin rather than CL-5 was reorganized as a consequence of IL-β treatment [32]. We previously demonstrated that once TJs form in HDMEC, the barriers are no longer sensitive to manipulations that specifically disrupt VE-cadherin interactions [16]. Thus it seems likely that the difference in the observed responses depend on whether AJs or TJs are being targeted. Since TJs provide a distinct contribution to the barriers formed by capillaries (as distinguished from venules) in many different vascularized organs, the pathway we have described in human ECs that leads to TJ disruption is thus more likely to be relevant to understanding the EC-intrinsic capillary component of organ failure in SIRS and severe sepsis [33].

We noted that CL5 was displaced but not degraded at TJs disrupted by TNF. In contrast to our findings in HDMECs, others have described rapid CL5 degradation in bovine retinal EC cultures treated with TNF [34] a finding that could be an early manifestation of the sensitivity of bovine ECs to TNF-mediated apoptosis observed in the absence of protein synthesis inhibitors [35]. Our data extends the observation that CL5 displacement without degradation by TNF occurs in HUVEC [36] to a human EC type with TJ-dependent barriers. We also noted that in a ROCK-dependent manner, TNF caused a reduction in the extent to which CL5 co-localized with cortical actin filaments. In assembled epithelial monolayers MLC phosphorylation suffices to trigger morphological changes in TJ structure associated with a loss of co-localization with perijunctional actin [37]. Thus, our data would suggest the linkage of CL5 to the actin cytoskeleton, believed mediated by ZO-1 and -2, is lost during TNF leak.

Our data show ROCK is activated downstream of NF-κB activation and that both responses contribute to phase 1 TNF leak. In support of these conclusions, genetic and pharmacologic methods of inhibiting TNF-induced activation of NF-κB or ROCK-1 and -2 each suffice to inhibit MLC phosphorylation, loss of cortical actin filaments, TJ disruption and leak, and at concentrations effective at inhibiting TNF-induced MLC phosphorylation, H-1152 and Y-27632 did not decrease NF-κB-dependent induction of ICAM-1. TNF-induced ROCK-dependent MLC phosphorylation appears to be independent of myosin phosphatase, because siRNA silencing of MYPT1 expression did not inhibit leak or TNF-induced MLC phosphorylation. Corroborating the latter point, TNF-induced ROCK activity (as measured by increased MLC phosphorylation) is still inhibited by Y-27632 in MYPT1 KD HDMEC. ROCK may instead directly phosphorylate MLC via the alternative pathway described by Amano et al [38]. In human lung microvascular EC, siRNA KD of both ROCK isoforms was more effective at inhibiting TNF-induced MLC phosphorylation than was individual knockdown of either ROCK-1 or -2 [26]. Previous reports also have described the role of ROCK in TNF-mediated EC leak as dispensable in bovine pulmonary artery EC [35], dispensable for late leak in HUVEC cultures [36], and required (ROCK-1, not-2 and the effect of dual ROCK-1/2 KD was not tested) for early (but dispensable for late) leak in human lung microvascular EC cultures [26], differences suggestive that TNF-induced ROCK activation varies by EC type. It is important to note that earlier studies did not describe distinct phases of TNF leak. In the present study, we have shown that the requirements for ROCK-1 and -2 activity differ in distinct phases of leak, something that could only be revealed by use of continuous ECIS monitoring. Conventional macromolecular flux measurements are simply too slow to resolve the dynamics of this process. Furthermore, TEER assessment by ECIS correlates inversely with macromolecular flux in HDMEC [17], consistent with similar observations by others who either disrupted or strengthened EC junctions [24,39–41].

siRNA knockdown of both ROCK isoforms showed that the contributions of ROCK-1 and -2 activity are additive and that phase 1 but not phase 2 leak is ROCK-dependent. Although the TEER decrease until the nadir of phase 1 (circa 2 h) was largely inhibited by combined siRNA knockdown of ROCK-1 and ROCK-2 expression, the subsequent TEER decrease from the interphase plateau until the phase 2 nadir (through 8 hours TNF) was not. In contrast, two different pharmacologic reagents widely regarded and commercially distributed as ROCK specific inhibitors effectively inhibited both nadirs of leak. That knockdown of ROCK-1 and -2 suffices to block loss of CL5/actin co-localization suggests that actin changes deriving from MLC phosphorylation are mostly relevant to phase 1 and not phase 2 TNF leak. We considered if the distinct effects of Y-27632 and H-1152 on phase 2 leak could derive from the very small amount of residual ROCK expression not silenced by siRNA. This seems unlikely because no inhibition of phase 2 leak was detected following highly effective knockdown of ROCK-1 and -2 expression. We therefore conclude that the inhibition of phase 2 leak by the serine/threonine kinase inhibitors H-1152 and Y-27632 likely derives from inhibition of different TNF-activated ser/thr kinases. The concentrations used here are comparable to their previous use on EC as ROCK-specific inhibitors in publications involving Y-27632 [35,36,42–44] and H-1152 [45–47]. Although early characterizations of Y-27632 [48,49] and H-1152 [50] showed ROCK selectivity, only a limited number of enzymatic targets were tested. Different studies show that at similar concentrations additional serine/threonine kinase substrates, namely serine/threonine-protein kinase N2 (PKN2; also referred to as protein-kinase C-related kinase 2 or PRK2) and leucine-rich repeat kinase 2 (LRRK2), are inhibited by Y-27632 and H-1152 [51,52].

**Fig 10:**
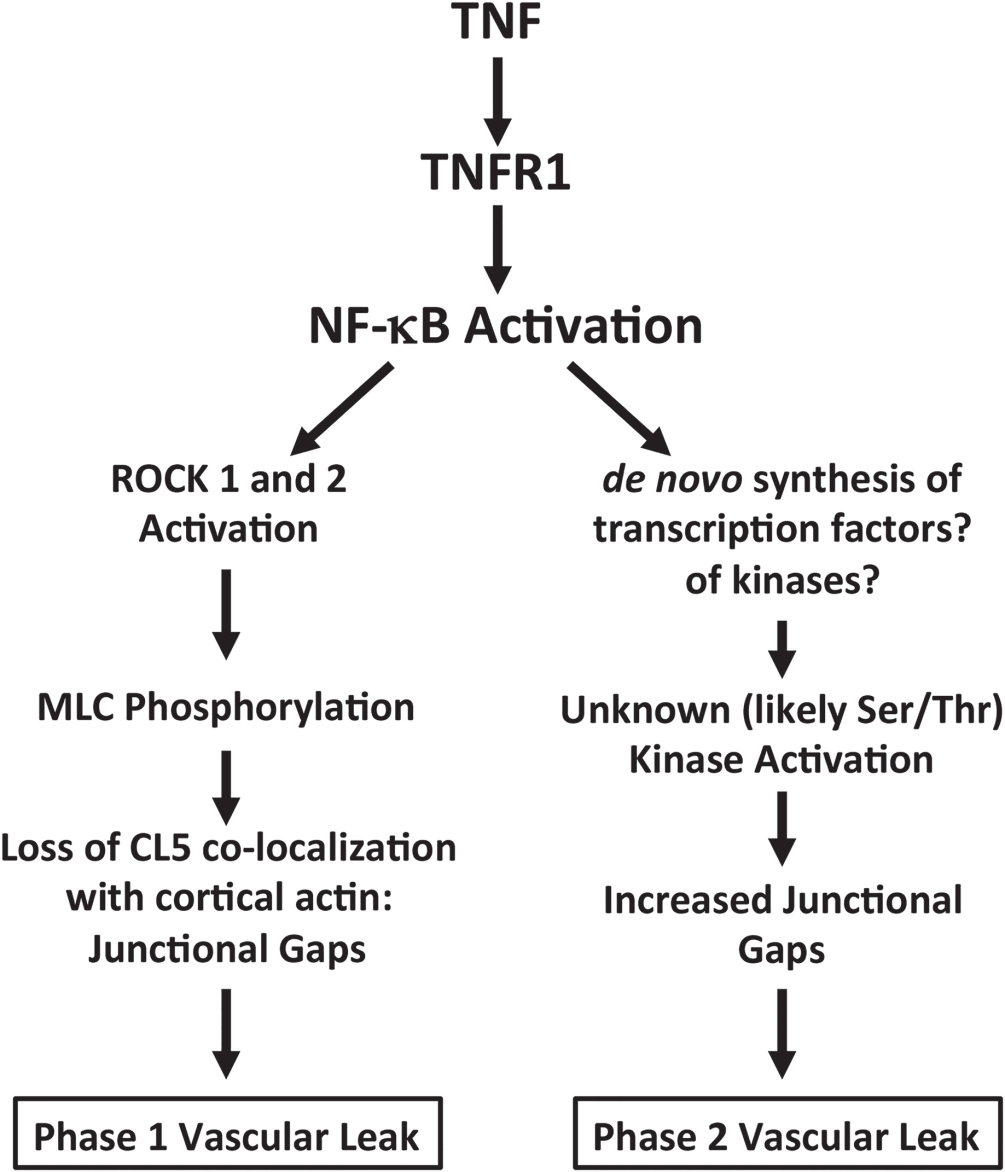
A schematic diagram illustrating the discrete steps of two different pathways mediating TNF phase 1 and phase 2 leak. Each arrow in this schematic diagram may involve multiple different steps.

In summary, we have investigated the effect of TNF on TJ-dependent human microvascular EC barriers that are organized around CL5. We report for the first time that such a process takes place in discrete steps, and by the failure of ROCK siRNA to reduce phase 2, that prevention of capillary leak may require targeting more than one pathway (Fig. 10). The conclusions drawn from these experiments on human cells begin to suggest strategies for preventing disruption of the TJs specifically found in the capillary (and not the post-capillary) segment of failing organs in SIRS or septic patients. Finally, although we have focused on changes that presumably occur acutely in continuous capillary beds, the NF-κB and ROCK-dependent mechanisms we discuss here may also be relevant to atherosclerotic changes in arteries where the inspissation of lipoproteins into the vessel intima that drives atheroma formation involves a similar disruption of endothelial TJs [53].
